# Pembrolizumab-Induced Myasthenia Gravis: A Case Report and Review of the Literature

**DOI:** 10.7759/cureus.41087

**Published:** 2023-06-28

**Authors:** Buse Eglenen Polat, Danish Safi, Maria Hafez, Amir Kamran

**Affiliations:** 1 Internal Medicine, West Virginia University (WVU) Medicine Camden Clark Medical Center, Parkersburg, USA; 2 Hematology and Medical Oncology, West Virginia University School of Medicine, Morgantown, USA; 3 Hematology and Medical Oncology, Charleston Area Medical Center, Charleston, USA

**Keywords:** myasthenia from checkpoint therapy, neurologic toxicity of immunotherapy, immune mediated myasthenia gravis, immune check point inhibitor adverse effects, immune check-point inhibitor

## Abstract

Myasthenia gravis (MG) is one of the most common neuromuscular adverse effects of immune checkpoint inhibitors (ICIs) and can result in significant morbidity and mortality when it affects the bulbar and respiratory muscles. Diagnosing immune-related MG (irMG) is challenging due to its nonspecific presentation and high negativity rate for MG antibody markers. Patients, primary care providers, and emergency care providers should be educated about MG as a potential adverse effect of ICIs for timely diagnosis and intervention.

## Introduction

Neurologic adverse effects are seen in 1-5% of patients using immune checkpoint inhibitors (ICIs) [1,2]. Myasthenia gravis (MG), along with encephalitis and peripheral neuropathy, is one of the most common neurologic adverse effects of ICIs [1-3]. ICIs can cause immune-related neuromuscular toxicity, causing new-onset MG and exacerbating existing MG [4]. Overactivation of cytotoxic T cells against neuronal antigens cross-presented on tumor cells and subsequent cytokine response are thought to be the primary underlying mechanisms of ICI-induced MG [5]. The most common adverse effects associated with ICI treatment are rash, colitis, thyroiditis, hypophysitis, hepatitis and pneumonitis [2], which can occur separately but tend to co-occur with each other. MG is commonly seen concurrent with immune-related myositis in about 30% of cases or myocarditis in about 8% of the cases, which are referred to as MG-myositis or MG-myocarditis overlap syndromes [2,6].

It is critical to recognize and treat immune-related MG (irMG) promptly since respiratory failure can develop secondary to respiratory and bulbar muscle involvement, which has been reported in up to 37% to 50% of the cases [3,6]. Mortality in irMG cases is most likely secondary to respiratory muscle involvement or myocarditis, and was reported in about 20% of the cases [3,6]. Overlap syndromes with myocarditis and myositis were associated with higher mortality compared to isolated irMG [3]. Diagnosing irMG is challenging as patients usually present with nonspecific neurological symptoms and the frequent absence of typical MG markers such as acetylcholine receptor (AChR) antibodies or anti-muscle-specific tyrosine kinase (MuSK) antibodies.

The onset of ICI-induced myasthenia typically occurs within the first couple of months following ICI initiation, with a median of four to six weeks [1-3,6]. The presence of AChR antibodies or, less commonly, anti-MuSK antibodies can aid in establishing the diagnosis. However, the absence of these antibodies does not rule out ICI-induced MG [3,7]. In fact, AChR antibodies are negative in approximately one-third of ICI-induced MG cases, compared to only 6% in idiopathic MG [3,8]. A characteristic electromyography (EMG) pattern with decrease of muscle action potential with repetitive nerve stimulation can help diagnose ICI-induced MG in the absence of autoantibodies [7]. While elevated creatine phosphokinase (CPK) is common in ICI-induced MG, it is considered a sign of myositis overlap in some reports [3,9]. The first-line treatment for suspected ICI-induced MG involves withholding ICI and initiating steroid therapy. Additionally, acetylcholinesterase inhibitors, intravenous immunoglobulin (IVIG), or plasma exchange can be used depending on the patient's and physician's preferences and the severity of symptoms [3,6]. One study reports better outcome when plasma exchange (PLEX) or IVIG were added to the treatment in early course compared to glucocorticoid treatment alone [3]. Most patients remain on slow taper of glucocorticoids or maintained on steroid-sparing agents such as azathioprine or mycophenolate mofetil as a part of long-term management [6]. 

Here we present a complex case of myasthenia gravis, concurrent with myositis, that had to be treated with high-dose steroids. Informed consent to publish this case was obtained from the patient.

## Case presentation

A 19-year-old female patient with a past medical history of congenital solitary kidney and stage III renal cell carcinoma (RCC) of the left kidney presented with dysphonia and dysphagia, persisting for three days. The symptoms gradually worsened throughout the day and were most pronounced in the evening. The patient was receiving a 200 mg IV infusion of pembrolizumab every 21 days for the treatment of her RCC. The onset of dysphonia and dysphagia was nine days after the second cycle of pembrolizumab (Day 30 after initiation of pembrolizumab treatment). After the first cycle of pembrolizumab, she had experienced thyroiditis and transaminitis.

**Figure 1:**
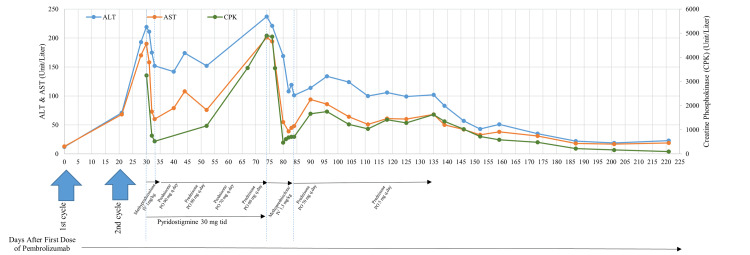
The trends of ALT (blue), AST (orange), and CPK (green) throughout the treatment. The primary Y axis represents ALT and AST in units/L, and the secondary Y axis CPK in U/L. The patient received pembrolizumab (200mg IV infusion) on Day 1 and Day 21 (blue arrow). The patient was hospitalized between Days 30-33 (blue dashed lines). Treatment with methylprednisolone (1mg/kg, IV) was started on Day 30 and was changed to prednisone 90mg PO per day during the discharge on Day 33 (black horizontal arrow). Pyridostigmine 30mg tid was started on Day 31 and continued until Day 74 (black horizontal arrow). Prednisone was tapered down, as shown. The patient was again hospitalized between Days 74-84 and received methylprednisolone (1.5mg/kg, IV) during hospitalization. The patient was discharged on prednisone 70mg PO per day and tapered down as shown. ALT: alanine transaminase, AST: aspartate aminotransferase, CPK: creatine phosphokinase, IV: intravenous, PO: orally, tid: three times a day

The initial workup revealed significant findings, including an elevated CPK level of 3,251 U/L (Normal 25-190 U/L) and an elevated aldolase level of 49.9 U/L (Normal: < 8.1 U/L). Persistent transaminitis and hypothyroidism were also noted in the blood work. Patient was asymptomatic with transaminitis and computed tomography (CT) of abdomen and pelvis did not show any abnormalities except for liver size at the upper limit of normal. During the admission thyroid-stimulating hormone (TSH) was elevated at 0.079 uIU/mL (Normal: 0.43-3.55 uIU/ml) and T4 was 0.63 ng/dL (Normal: 0.70-1.25 ng/dL). CT scan of the head and neck showed no acute pathologies, such as acute intracranial ischemia or hemorrhage. A laryngoscopy of the vocal cords, performed by an otolaryngologist, showed no damage or dysfunction. AChR, MuSK, and Anti-Jo1 (anti histidyl transfer RNA [t-RNA] synthetase) antibodies were negative. Electromyography (EMG) with repetitive nerve stimulation could not be performed due to the patient's intolerance to the procedure. The diagnosis of irMG was clinically established, considering the involvement of small muscles and muscle fatiguability. Although an overlap syndrome cannot be excluded in this case, myositis was not considered as the primary diagnosis due to the lack of typical symptoms associated with myositis, such as weakness of proximal muscles and myalgias. Treatment was initiated with intravenous methylprednisolone at a dose of 1 mg/kg and oral pyridostigmine at a dose of 30 mg three times daily, resulting in subsequent symptom improvement. Liver enzymes and CPK levels trended down after the initiation of treatment (Figure 1).

The patient was discharged on the third day of admission and prescribed prednisone at a dose of 90 mg daily at discharge. The Naranjo Adverse Drug Reaction Probability Scale score for this case was 8, indicating probable causality between the medication and the adverse effect (Table 1).

**Table 1:** The Naranjo Adverse Drug Reaction Probability Scale. The Naranjo scale for the index case was 8, indicating probable adverse drug reaction (ADR). Interpretation of Naranjo Scale: ≥ 9 = definite ADR, 5-8 = probable ADR, 1-4 = possible ADR, 0 = doubtful ADR.

Question	Yes	No	Do Not Know	Score for the Index Case
1. Are there previous conclusive reports on this reaction?	1	0	0	1
2. Did the adverse event appear after the suspected drug was administered?	2	-1	0	2
3. Did the adverse event improve when the drug was discontinued or a specific antagonist was administered?	1	0	0	2
4. Did the adverse event reappear when the drug was readministered?	2	-1	0	0
5. Are there alternative causes that could on their own have caused the reaction?	-1	2	0	2
6. Did the reaction reappear when a placebo was given?	-1	1	0	0
7. Was the drug detected in blood or other fluids in concentrations known to be toxic?	1	0	0	0
8. Was the reaction more severe when the dose was increased or less severe when the dose was decreased?	1	0	0	0
9. Did the patient have a similar reaction to the same or similar drugs in any previous exposure?	1	0	0	1
10. Was the adverse event confirmed by any objective evidence?	1	0	0	0
				8

As the oral prednisone was tapered down with outpatient follow-up, the patient developed exertional shortness of breath associated with an elevation of troponins and a re-elevation of CPK, raising concerns for immune-mediated myositis and myocarditis. Consequently, the patient was readmitted on Day 74. During the admission, CPK levels peaked at 4902 U/L (normal range: 25-190 U/L), and troponin levels were measured at 1046 ng/L (normal range: 0-30 ng/L). Glucocorticoid treatment was initiated with intravenous methylprednisolone at a dose of 1.5 mg/kg, resulting in subsequent improvement of clinical symptoms and troponin and CPK levels. Troponin levels decreased to 66 ng/L, and CPK levels dropped to 710 U/L before the discharge. The patient was discharged with an additional taper of oral glucocorticoids. CPK levels continued to decrease during outpatient follow-up (Figure 1).

## Discussion

Here, we present a demonstrative case of irMG after using pembrolizumab and review previously published case reports (n=12) of de novo myasthenia gravis after pembrolizumab therapy (Table 2) [8,10-20]. In our literature review, the onset of symptoms related to ICI-related MG ranged between 14 and 90 days following the initiation of ICI, with an average of 43 days (± 33 days). This correlates with previous retrospective studies that reported symptom onset within one to 16 weeks, with an average of four weeks [3]. Our index case presented symptoms 30 days after ICI initiation.

**Table 2:** Case reports of immune-related myasthenia gravis are shown. The author and publication date, type of malignancy and type of immune checkpoint inhibitor, antibody positivity associated immune reactions, and treatment used are reported in the table. RCC: renal cell carcinoma, SCLC: small cell lung cancer, NSCLC: non-small cell lung cancer, MM: malignant melanoma, TCC: transitional cell carcinoma (urothelial cell carcinoma), HNSCC: head & neck squamous cell carcinoma, SQCLC: squamous cell carcinoma, AChR: acetylcholine receptor antibody, MuSK: muscle-specific tyrosine kinase inhibitor, AcHe: acetylcholinesterase, IVIG: intravascular immunoglobulin

Author	Type of Malignancy	Patient age	Symptom Onset	Markers			Associated Adverse Effects			Treatment		
				AChR	MusK	CPK	Transaminitis	Myocarditis	Myositis	Steroid	AChE inhibitor	IVIG
Hibino et al. [10]	SQCLC	83	38	Negative	Negative	Elevated	✔			✔	✔	
Alnahhas et al. [11]	MM	84	90	Positive	Negative	Normal						✔
Huh et al. [12]	Thymic cancer	34	56	Positive	N/A	Elevated			✔	✔		✔
Gonzalez et al. [13]	Osteosarcoma	71	N/A	Negative	Negative	Elevated					✔	
Hernandez et al. [14]	Thymoma	48	10	Positive	N/A	Elevated	✔	✔			✔	
Heleno et al. [15]	Colorectal	43	90	Positive	N/A	N/A					✔	
Botta et al. [16]	TCC	72	72	Positive	Negative	Elevated	✔			✔	✔	
Tian et al. [8]	TCC	75	16	Negative	Negative	Elevated	✔			✔		
Jenkins et al. [17]	SQCLC	65	N/A	Negative	Negative	Elevated	✔	✔		✔	✔	✔
Szuchan et al. [18]	Thymic cancer	70	21	Positive	Negative	Elevated		✔		✔	✔	
Hayakawa et al. [19]	TCC	84	25	Negative	Negative	Elevated			✔	✔		
Lara et al. [20]	NSCLC	63	N/A	Negative	Negative	N/A				✔	✔	✔

Among the reviewed cases, AChR antibodies were detected in six (50%) cases, and elevated CPK levels were reported in nine (75%) cases, consistent with previous reports (Table 2). In six (50%) of the reviewed cases, both AcHR and anti-MuSK autoantibodies were negative, while the CPK level was elevated (Table 2). In our case, AcHR and anti-MuSK antibodies were negative, and the diagnosis was made clinically based on the history of small muscle involvement, muscle fatiguability, and elevated CPK and aldolase levels. While EMG findings can confirm the diagnosis of MG, EMG may not be readily available, time-consuming, and not well-tolerated by patients. One of the major limitations of our case is lack of EMG for definite diagnosis of MG and ruling out myositis. 

ICI-related MG can co-occur with myositis, myocarditis, thyroiditis, and transaminitis/hepatitis, which may further complicate the course (Table 2). Among the reviewed case reports, eight (66%) reported concurrent immune-related adverse effects (irAE) (Table 2). Most commonly, five (41.6%) patients had transaminitis in addition to ICI-related MG. Our index case presented transaminitis, thyroiditis, and myocarditis in addition to irMG.

The most preferred treatment among the reviewed cases was oral or IV steroids, used in eight (66%) cases, and acetylcholinesterase inhibitors, used in eight (66%) cases (Table 2). IVIG treatment was added in cases where steroid treatment failed [6]. Our case has been treated with steroids and acetylcholinesterase inhibitors, resulting in the resolution of symptoms.

IrMG should be considered in patients presenting with neurological symptoms up to 16 weeks after initiating ICIs. The workup for irMG includes AcHR and anti-MuSK antibodies and muscle enzymes, although their absence does not rule out irMG. Clinical symptoms at presentation should be prioritized in the diagnosis. Withholding the ICI is usually the first step in management.

## Conclusions

Timely diagnosis of ICI-induced MG and other neurologic toxicities associated with ICIs is essential, as the involvement of respiratory or bulbar muscles can require intubation and cause mortality in the affected patients. A high clinical suspicion is necessary for the diagnosis of ICI-related MG. MG can be seen up to 16 weeks after ICI use. Although MG-associated antibodies can help establish the diagnosis, they can be negative in one-third of the cases. EMG helps establish the diagnosis when available. Mainstay treatment is withholding ICI treatment and initiation of glucocorticoid treatment.
